# Dysphagia Secondary to Myotonic Dystrophy Unveiled in a Case of Destructive Spondylitis With Synovitis, Acne, Pustulosis, Hyperostosis, Osteitis (SAPHO) Syndrome Presenting As Torticollis

**DOI:** 10.7759/cureus.54271

**Published:** 2024-02-15

**Authors:** Yoshinori Ishikawa, Takashi Kobayashi, Ryo Shoji, Naohisa Miyakoshi

**Affiliations:** 1 Orthopedic Surgery, Akita Kousei Medical Center, Akita, JPN; 2 Orthopedic Surgery, Akita University Graduate School of Medicine, Akita, JPN

**Keywords:** myotonic dystrophy, post-anesthetic dysphagia, sapho syndrome, destructive spondylitis, adult torticollis

## Abstract

To report an instructive case involving destructive spondylitis and synovitis-acne-pustulosis-hyperostosis-osteitis (SAPHO) syndrome, presenting with torticollis and postoperative dysphagia without hoarseness, attributed to hidden myotonic dystrophy (DM).

A 51-year-old male patient with a cervical deformity, who was previously managed conservatively for a metastatic tumor, underwent reconstruction surgery and subsequently experienced postoperative dysphagia. The presence of destructive spondylitis with torticollis, warranting prompt assessment to prevent paralysis, adds complexity to the delayed identification of DM. Given the rarity of DM, peculiar neurological symptoms and other systemic comorbidities did not lead to a preoperative diagnosis without prior knowledge.

The patient’s dysphagia induced respiratory arrest and required reintubation. Challenges in extubation and ventilator weaning arose due to hypercapnia. Superimposed COVID-19 infection elongated the duration of intubation. Extubation failed due to aspiration pneumonia and required a tracheotomy. Despite laryngeal elevation and preservation of the relaxation of the oesophageal entrance, the sensation and movement of the tracheopharynx were disturbed. The patient exhibited an oropharyngeal propulsive disorder, predominantly indicative of motor neuron disease. The patient’s mother stated that his brother had been hospitalized for a long time after abdominal surgery. Finally, the patient was diagnosed with DM, which is known to cause post-anesthetic dysphagia.

Recognizing the existence of severe destructive cervical spondylitis associated with SAPHO is crucial. Although DM is not very common, it is not classified as extremely rare. Therefore, surgeons should be mindful of the potential risks associated with general anesthesia in patients with DM. The complexity of preoperative conditions may hinder an accurate diagnosis. Recognizing and establishing preoperative expectations can assist surgeons in preventing complications, even if complex spinal surgery is required for patients with DM.

## Introduction

The SAPHO syndrome is characterized by synovitis, acne, pustulosis, hyperostosis, and osteitis. Its prevalence is estimated to be 1 in 10,000 in Caucasian populations [1,2]. Destructive cervical spondylitis with SAPHO syndrome is rare; however, awareness of a potential cervical deformity with SAPHO syndrome mimicking tumor-like lesions should be promoted. Surgeons should also recognize radiographic characteristics different from those of pyogenic spondylitis. An unusual postoperative course of dysphagia, aspiration pneumonia, and tracheotomy was revealed to be the result of overlooked myotonic dystrophy (DM). DM is a rare autosomal dominant disorder, with an estimated prevalence of 8-10 per 100,000 individuals [3,4]. However, a recent study suggested a rate of 1 in 2,100 individuals [5]. Therefore, it is not extremely rare, and it would be possible to encounter patients with DM requiring spinal surgery. Surgeons should be aware preoperatively that patients with DM may experience difficulties in extubation due to dysphagia after general anesthesia. In this context, we present an annotated but instructive case of destructive spondylitis representing torticollis with SAPHO syndrome and post-anesthetic dysphagia caused by DM.

## Case presentation

**Figure 1:**
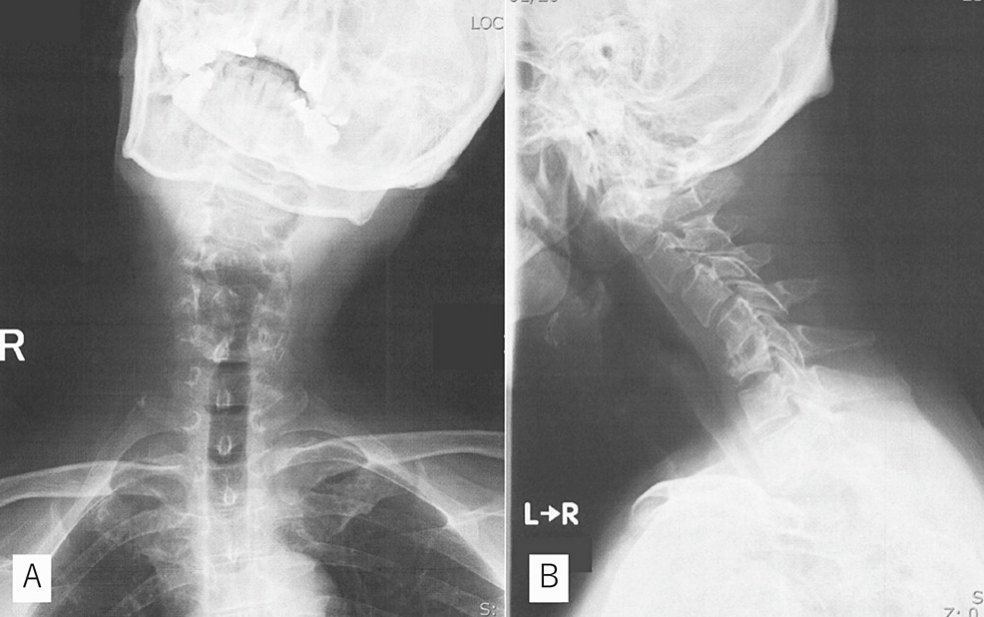
Preoperative X-ray X-ray showing flexion-rotation deformity (A) and wedge deformity at C6 (B).

A 51-year-old male with atrial fibrillation (AF), sleep apnoea syndrome (SAS), and untreated diabetes experienced progressive neck pain for two months. The family doctor diagnosed the patient with a metastatic tumor; however, he was treated conservatively for unknown reasons. He was referred to us because he relocated to our area. He presented with torticollis but could walk without support. His body temperature was recorded at 37.0℃. Physical examination revealed no motor or sensory deficits and no evidence of hyperreflexia in the four extremities. The Hoffman and Romberg signs were both negative. However, the bilateral 10-second grasp and release test exhibited deterioration 15/12 times. Radiographs revealed flexion-rotation deformity and wedge deformities at C6 (Figure 1).

**Figure 2:**
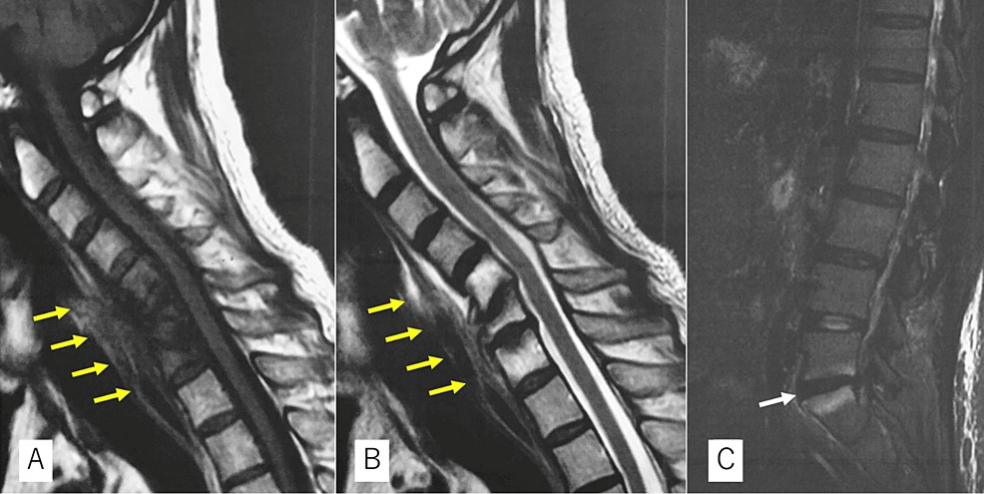
Preoperative MRI scan Magnetic resonance image showing low signal intensity with T1-weighted images (A) and high signal intensity with T2-weighted images (B) from C5 to C7 with retropharyngeal swelling (yellow arrows). The L5-S1 segment also showed a high signal intensity change in the short tau inversion recovery (STIR) image without disc involvement (white arrow) (C).

**Figure 3:**
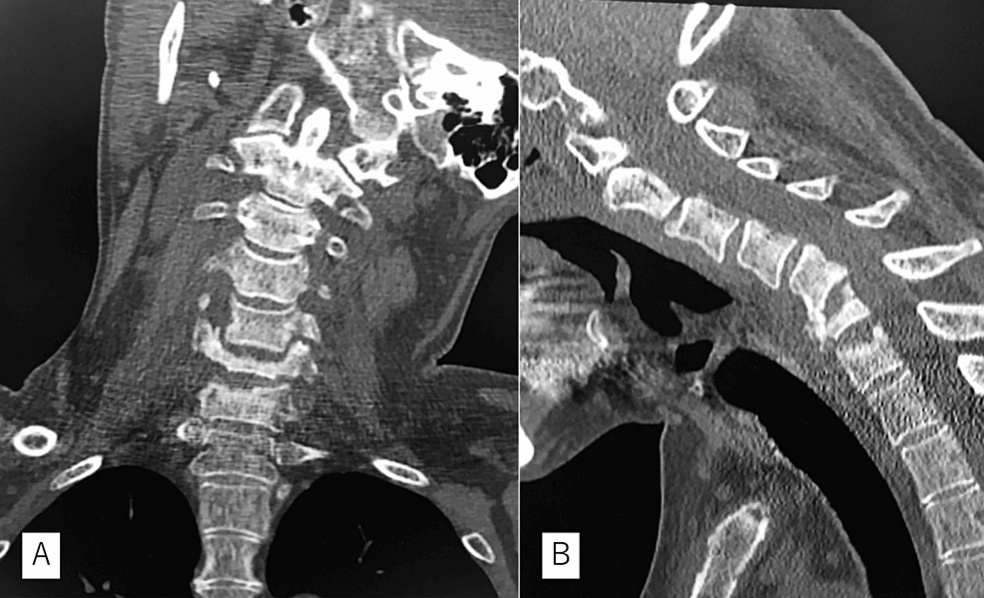
Preoperative CT scan Computed tomography revealing rotational deformity with coronal image (A) and destructive wedge deformity within sclerotic change (B).

Magnetic resonance imaging (MRI) revealed low signal intensity with T1-weighted images and high signal intensity with T2-weighted images from C5 to C7 with retropharyngeal swelling (Figures 2A-2B). Computed tomography (CT) revealed a destructive deformity within the sclerotic changes (Figure 3) and no apparent tumor origin on whole-body scans. Blood examination revealed slightly elevated C-reactive protein (1.7 mg/dL), 6900 white blood cells per mm^3^ (69.0% neutrophils and 19.6% lymphocytes), and negative tumor markers with no bacteremia on blood culture. Coexisting lumbago and MRI revealed an L5-S1 intensity change without disc involvement, suggesting axial spondyloarthritis (Figure 2C). However, he did not mention any family history of spondyloarthritis or any symptoms related to bowel, eye, or sacroiliac. Therefore, we could not deny pyogenic spondylitis or metastatic tumors.

**Figure 4:**
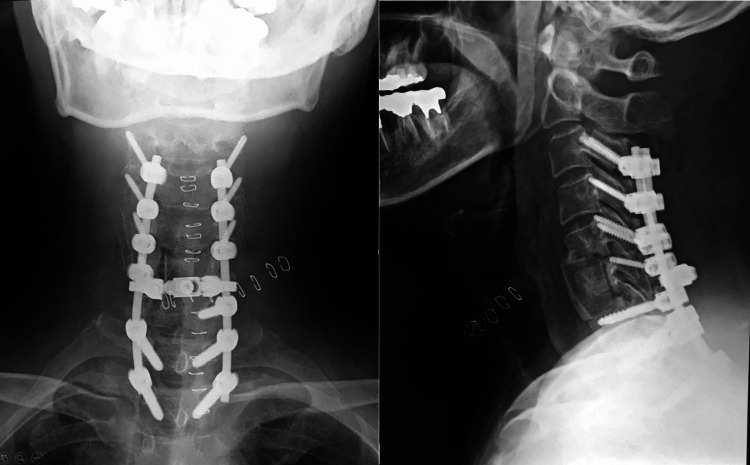
Postoperative X-ray C6 corpectomy with iliac bone graft and posterior spinal fusion from C3-T2 was performed while taking care to avoid hyperlordosis so as to prevent postoperative dysphagia and C5 palsy.

Planning for the reconstruction surgery took into account the need to avoid hyperlordosis to minimize the risk of postoperative dysphagia and C5 palsy. A C6 corpectomy utilizing an iliac bone graft and posterior spinal fusion from C3-T2 was performed (Figure 4).

**Figure 5:**
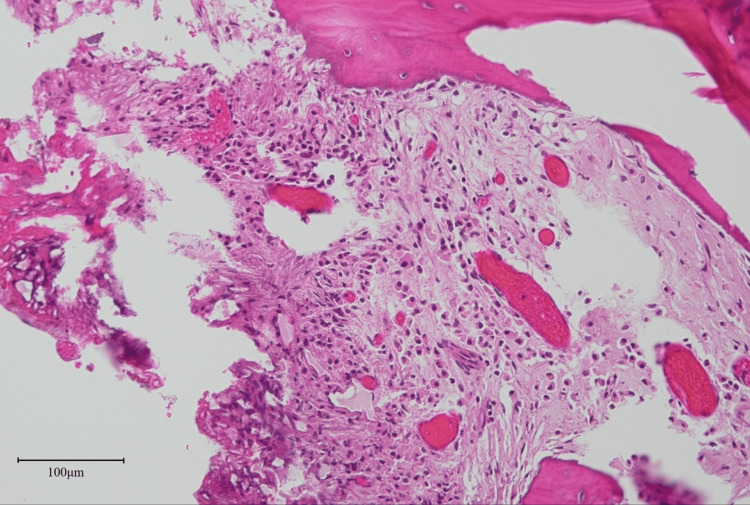
Histological examination Pathological examination revealing no malignant cells and showing chronic infection with the infiltration of neutrophils into the destroyed bone marrow (haematoxylin and eosin staining ×20).

The following day, the patient experienced difficulty swallowing without hoarseness. The patient did not show hypoxia, but his mouth was filled with saliva. Consultations with otolaryngologists were postponed due to their unavailability. At midnight, the patient suffocated with saliva and experienced respiratory arrest. Emergency intubation reestablished the patient’s breathing. The absence of a gag reflex during ventilator control raised suspicions of neurological causes, as indicated by the anesthesiologist. The patient had hypercapnia and did not show spontaneous breathing during ventilator weaning. SAS was suspected for the cause of hypercapnia. Around that time, pathological examination revealed no malignant cells and a suspected chronic infection was identified with infiltration of neutrophils into the bone (Figure 5).

We suspected axial spondyloarthritis once again and found a skin rash on him. A dermatologist diagnosed palmoplantar pustulosis and SAPHO syndrome. During delayed extubation, the patient was infected with COVID-19. The hospital director prohibited extubation to prevent COVID-19 transmission to other patients, and after 12 days, an attempt at extubation was made. However, aspiration pneumonia was also observed. A subsequent bronchoscopy, performed by an otolaryngologist, revealed poor sensory function and deteriorated movement of the tracheopharynx, while laryngeal elevation and the relaxation of the oesophageal entrance were preserved. Although they were not initially recognized, the patient showed symptoms of oropharyngeal propulsive disorder. A tracheotomy was performed to prevent aspiration pneumonia. Although the patient displayed acceptable cervical alignment without hyperlordosis or narrow retropharyngeal space, the cause of dysphagia remained unknown. Therefore, we began to consider reducing lordosis via reoperation. At the same time, we suspected motor neuron disease (MND). The patient was strongly suspected of having DM only when the patient’s mother revealed that the patient’s brother had been hospitalized for MND after abdominal surgery in a subsequent visit. The patient was taken off the ventilator but could not remove the tracheal cannula due to silent aspiration and had to continue tube feeding. Cervical reconstruction was not performed. The patient was transferred to a specialized hospital for MND, where a genetic examination confirmed the definitive diagnosis.

## Discussion

SAPHO syndrome is diagnosed in the presence of any of the following: osteoarticular manifestations of severe acne, palmoplantar pustulosis, hyperostosis, or chronic recurrent multifocal osteomyelitis [6]. SAPHO syndrome with destructive cervical spondylitis is rare, and only four studies report surgical reconstruction in the English literature [7-10]. Moreover, the occurrence of cervical deformities, specifically torticollis, in association with SAPHO syndrome has not been previously reported. Therefore, it was difficult to recognize SAPHO syndrome-related destruction, which caused postoperative delayed diagnosis. The extensive damage to the vertebrae not only complicates the precise diagnosis but also increases the risk of misdiagnosis, often resembling metastasis. Various combinations of vertebral marrow edema and sclerosis, destructive lesions of the vertebral body, and collapse mimic neoplastic diseases. Therefore, it is crucial to recognize characteristic radiological findings that typically start from the vertebral endplate or enthesis to the surrounding soft tissue without disc involvement [11]. Our patient exhibited no disc involvement in the lumbar lesion on the short tau inversion recovery (STIR) sequence of the MRI (Figure 2C). Precise radiographic interpretation may be related to an early and correct diagnosis. Skin lesions are one of the most characteristic manifestations of SAPHO syndrome, and more than 60% of patients demonstrate the cutaneous lesions required for a definitive diagnosis [2,12]. In SAPHO-associated spondylitis, medical therapy is the first choice in the absence of spinal instability or myelopathy.

DM is characterized by myotonia, muscle weakness, and cataracts along with systemic disorders such as respiratory, swallowing, cardiac, metabolic, and higher brain dysfunction. In our patient, the presence of AF, SAS, diabetes, and grip myotonia that mimicked clumsy hands were notable and related to DM. DM might also aggravate cervical deformity with SAPHO syndrome. The characteristic appearance of a hatchet face, frontal baldness, or the presence of inappropriate multiple comorbidities relative to the patient’s age may prompt experienced doctors to consider DM. However, anesthesia often triggers a postoperative respiratory failure with hypercapnia, making a preoperative diagnosis of DM crucial. Opioid use is reported as the main cause of respiratory failure and can result in pharyngolaryngeal muscle weakness and dysphagia [13,14]. In the present case, the otolaryngologist noted muscle weakness when the patient swallowed during bronchoscopy. The presence of oropharyngeal propulsive disorder usually raises suspicions of MND, however, the existence of MND was not noticed at that time. Moreover, postoperative dysphagia may have been attributed not to cervical alignment but to opioid use, given the simultaneous occurrence of hypercapnia and pharyngeal muscle weakness. The prevalence of DM is far greater than we imagined, and it is possible that we will encounter it in practice. Surgeons performing cervical reconstruction should possess a minimum level of knowledge regarding the swallowing problems associated with MND. In this case, the patient’s mother did not recognize DM because of the complexity of MND. Therefore, family history should be noted carefully and in detail several times if MND is strongly suspected.

Destructive spondylitis is typically caused by pyogenic spondylitis or metastatic tumors, and it can lead to severe cervical deformity. This deformity needs prompt stabilization to prevent spinal cord injuries. However, it is essential to note that SAPHO-related spondylitis can also cause destructive cervical spondylitis and should be considered in the differential diagnosis. Furthermore, we should be aware of atypical physical findings that may indicate peculiar diseases. This will help us perform all necessary examinations for preoperative diagnosis, especially in patients with DM.

## Conclusions

The potential for destructive cervical spondylitis in SAPHO syndrome has not been fully recognized to date. Earlier awareness could prompt consideration of other distinctive symptoms, such as grasping myotonia and systemic diseases, though it proved unsuccessful in our case. While the risks of general anesthesia in patients with MND are well known, the complexity of preoperative conditions can make it challenging to diagnose DM in time. Only knowledge and experience enable the successful treatment of rare diseases that require complicated spine surgery.
